# Five new species of the genus *Euplocania* Enderlein (Psocodea, ‘Psocoptera’, Psocomorpha, Ptiloneuridae) from Colombia

**DOI:** 10.3897/zookeys.711.20683

**Published:** 2017-10-23

**Authors:** Ranulfo González-Obando, Alfonso N. García Aldrete, Nancy S. Carrejo

**Affiliations:** 1 Departamento de Biología, Facultad de Ciencias Naturales y Exactas, Universidad del Valle, Santiago de Cali, Colombia; 2 Departamento de Zoología, Instituto de Biología, Universidad Nacional Autónoma de México, Apdo. Postal 70-153, México, D. F., México

**Keywords:** Neotropics, South America, Taxonomy

## Abstract

Five new species of *Euplocania* from Colombia belonging in four species groups are here described and illustrated. They increase to 22 the number of species described in the genus, thirteen of which are endemic to Colombia, with one species shared with Brazil and Peru. Three new species groups of *Euplocania* are here diagnosed. A key to the identification of males of Colombian *Euplocania* is included.

## Introduction

*Euplocania* Enderlein (1910) presently includes fifty six species, 16 of which have been described (García Aldrete et al. 2013; González-Obando et al. 2015). Nine species of this genus are known in Colombia (Table 1), as well as an undetermined fossil species in Quaternary copal (Azar et al. 2009; García Aldrete et al. 2013; González-Obando et al. 2015).

**Table 1.** List of Colombian species of *Euplocania* Enderlein, groups and distribution.

Species	Group	Department
*E. badonneli* New & Thornton*	*Amabilis*	Amazonas, Caquetá, Putumayo
*E. bonaverensis* González, García Aldrete & Carrejo	*C*	Valle del Cauca
*E. caliensis* González, García Aldrete & Carrejo	*A*	Valle del Cauca
*E. caquetaensis* sp. n.	*B*	Caquetá
*E. daguaensis* González, García Aldrete & Carrejo	*B*	Valle del Cauca
*E. danubiana* González, García Aldrete & Carrejo	*E*	Valle del Cauca
*E. gaitanae* sp. n.	*B*	Caquetá
*E. guentherbuchi* González, García Aldrete & Carrejo	Guentherbuchi**	Huila
*E. laelsa* sp. n.	Laelsa**	Valle del Cauca
*E. macarenaensis* González, García Aldrete & Carrejo	*Amabilis*	Meta
*E. nasa* sp. n.	Guentherbuchi**	Huila
*E. reyesi* García Aldrete, González & Carrejo	*Zelayensis*	Magdalena
*E. vallecaucana* González, García Aldrete & Carrejo	*C*	Valle del Cauca
*E. yalcona* sp. n.	Yalcona**	Huila

* Distribution: Peru (Madre de Dios), Brazil (Rondônia), Colombia. ** New group

Specimens collected in the framework of the project “Revisión Taxonómica y Endemismo de los Psócidos (Insecta: Psocodea: ‘Psocoptera’) de Areas Protegidas de Colombia”, financed by Colciencias-Universidad del Valle, increase this number to 31 species, 22 of them undescribed. García Aldrete et al. (2013) and González-Obando et al. (2015) proposed several species groups in the genus. The purpose of this work is to describe and illustrate five new species that belong in one of those groups (B) and to define three additional groups that are diagnosed here. The species here described were found in natural areas of three Colombian departments, one in Valle del Cauca (western slope of the western mountain range), two in Huila (central cordillera), and two in Caquetá (eastern slope of the eastern cordillera, within the Amazon Basin).

## Materials and methods

Ten males and four females were available for study. They belong to the collection of the Group of Entomological Investigations (Departamento de Biología, Facultad de Ciencias Naturales y Exactas, Universidad del Valle, Santiago de Cali, Colombia), and are deposited in the Entomological Museum of the Universidad del Valle (MUSENUV), Santiago de Cali, Colombia.

One male and one female of each species were dissected in 80% ethanol, and their parts (head, right wings and legs and terminalia), were mounted on slides in Canada balsam, following standard procedures. Color was recorded by placing whole specimens, before dissection, under a stereoscopic microscope, illuminated with cold, white light at 50×. Parts on the slides were measured, following standard procedures, and the measurements are given in mm; the illustrations were made from digital photographs, taken with a Canon T5i camera and Helicon Focus program, processed in a vector graphics editor Clip Studio Paint.

Abbreviations of parts measured are as follows:

**al/ah** areola postica length/height

**ctt_1_** number of ctenidiobothria on t_1_

**f_1_-f_n_** lengths of flagellomeres 1-n of right antenna

**F, T, t_1_-t_3_** lengths of femur, tibia, and tarsomeres 1-3 of right hindleg

**FW** and **HW** lengths of fore- and hind wings

**IO, D**, and **d** minimum distance between compound eyes, antero-posterior diameter and transverse diameter, respectively, of right compound eye, all in dorsal view of head

**L/W** forewing length/forewing width

**lp/wp** pterostigma length/pterostigma width

**l/w** hindwing length/hindwing width

**Mx4** length of fourth segment of right maxillary palpus

**MxW** maximum width of head capsule in frontal view

**PO** d/D, H: head length (in dorsal view)

## Results

### Key to males of Colombian *Euplocania*.

(*E. macarenaensis* González et al., of which males are unknown, is not included)

**Table:** 

1	Forewings mostly hyaline, with slender pigmented band from R_4+5_ to cell Cu_2_, pterostigma rounded, not extended to Rs, veins with brown areolae at insertion of setae. Hypandrium of one sclerite, deeply cleft in the middle	**Group D**...**2**
–	Forewings variable; hypandrium of three sclerites, the laterals separated from the central (Figs 4, 16, 28, 40)	**3**
2	Posterior projections of hypandrium with apices rounded (see fig. 67 in González-Obando et al. 2015). Phallosome with two pairs of endophallic sclerites, acuminate projections of anterior pair wide based (see fig. 68 in González-Obando et al. 2015)	***E. vallecaucana* González, García Aldrete & Carrejo**
–	Posterior projections of hypandrium dilated anteapically (see fig. 7 in González-Obando et al. 2015). Phallosome with a long, mesal, distally acuminate sclerite, in addition to the two pairs of endophallic sclerites; projections of the anterior pair slender, not wide based (see fig. 8 in González-Obando et al. 2015)	***E. bonaverensis* González, García Aldrete & Carrejo**
3	Forewings hyaline, or with a pigmented marginal band, from cell R_3_ to ends of cells Cu_2_ and 1A or to wing base; pterostigma long, slender; central sclerite of hypandrium with two or four tapered posterior projections (Figs 1, 13, 25, 37, 43)	**4**
–	Forewings with deeply pigmented, broad marginal band, from cell R_3_ or R_5_ to wing base; pterostigma distinctly projected towards Rs; central sclerite of hypandrium with two posterior projections variable in shape and position	**Groups Amabilis, A, E**...**11**
4	Central sclerite of hypandrium wide, posteriorly with four posterior projections, two broad lateral and two acuminated small, median (Figs 4 and 16). Forewings with pigmented marginal band, from cell R_3_ to near wing base, M four branched, M_4_ simple (Figs 1, 13)	**5**
–	Central sclerite of hypandrium with two-four posterior lateral projections, lateral ones acuminate (Figs 28, 40, 46). Forewings with or without marginal band, M four-five branched (Figs 25, 37, 43), if M four branched, then M_4_ simple or forked	**7**
5	Lateral projections of central sclerite of hypandrium with a blunt ended process, directed inwards, median projections separated from the base or from the distal portion (Fig. 16)	**6**
–	Lateral projections of central sclerite of hypandrium without a blunt ended process, with abundant distal teeth, some thick, median projections separated from the base (Fig. 4)	***E. caquetaensis* sp. n.**
6	Central sclerite of hypandrium with teeth on the inner side of the lateral projection, median projections separated from the base (see fig. 25 in González-Obando et al. 2015)	***E. daguaensis* González, García Aldrete & Carrejo**
–	Central sclerite of hypandrium without teeth on the inner side of the lateral projection, median projections separated from the distal portion (Fig. 16)	***E. gaitanae* sp. n.**
7	Forewings with a slender or broad pigmented band, M four branched, M4 simple (Figs 25 and 43). Central sclerite of hypandrium with two-four posterior acuminate projections, if four, two lateral and two median (Fig. 46), if two, lateral (Fig. 28)	**8**
–	Forewings almost hyaline, without pigmented marginal band (Fig. 37), M of four-five branches, M_4_ or M_5_ forked. Central sclerite of hypandrium with two posterior projections, broad or acuminate (Fig. 40)	**9**
8	Forewings with broad, marginal, pigmented band, from cell r2+3 to vein A1 (Fig. 25). Central sclerite of hypandrium with two lateral, broad convergent projections, apically overlapping, distally denticulate (Fig. 28)	***E. laelsa* sp. n.**
–	Forewings with narrow, marginal, pigmented band, from R_2+3_ to wing base (Fig. 43). Central sclerite of hypandrium with four acuminate projections, the median ones shorter and separated by a U-shaped concavity (Fig. 46)	***E. yalcona* sp. n.**
9	Posterior projections of central sclerite of hypandrium convergent, distally rounded and crossed (see García Aldrete et al. 2013). Phallosome with two mesal endophallic sclerites, clearly separated and distally acuminate (see fig. 5 in García Aldrete et al. 2013)	***E. reyesi* García Aldrete, González & Carrejo**
–	Posterior projections of central sclerite of hypandrium not convergent, broad or acuminate, never distally crossed (Fig. 40). Phallosome as in Fig. 42, with mesal endophallic sclerite fused, transverse, postero-mesal endophallic sclerites tapered and distally bent outwards	**10**
10	Central sclerite of hypandrium with slender posterior projection, with apex bent inwards (see fig. 49 in González-Obando et al. 2015). Mesal endophallic sclerites with a rounded protuberance in the middle of the bridge, each arm dilated mesally, curved outwards, distally acuminate (see fig. 50 in González-Obando et al. 2015)	***E. guentherbuchi* González, García Aldrete & Carrejo**
–	Central sclerite of hypandrium with posterior projections proximally wide (Fig. 40). Phallosome as in Fig. 42	***E. nasa* sp. n.**
11	Central sclerite of hypandrium with two posterior projections; side sclerites smaller than central sclerite	**12**
–	Central sclerite of hypandrium with four posterior projections, two central ones long, slender, glabrous, and two side ones broad, setose, with digitiform posterior processes bearing setae; side sclerites about twice as large as central sclerite (see fig. 37 in González-Obando et al. 2015). Phallosome with posterior, transverse endophallic sclerite, with posterior border projected in the middle (see fig. 38 in González-Obando et al. 2015)	***E. danubiana* González, García Aldrete & Carrejo**
12	Central sclerite of hypandrium with blunt ended lateral posterior projections, median concavity U-shaped; phallosome with mesal endophallic sclerite transverse but not W-shaped (see figs 23 and 24 in New and Thornton 1988)	***E. badonneli* New & Thornton**
–	Posterior projections of central sclerite of hypandrium median, arising from a common stem, each arm distally acuminate; phallosome with a transverse, mesal endophallic sclerite W-shaped (see figs 13 and 14 in González-Obando et al. 2015)	***E. caliensis* González, García Aldrete & Carrejo**

### Taxonomy

#### Euplocania caquetaensis sp. n.

Taxon classificationAnimaliaPsocodeaPtiloneuridae

http://zoobank.org/97DD41A4-943B-4A49-99C0-4F2201051E55

Figs 1–6 , 7–12

##### Type locality.

COLOMBIA. Caquetá. San Vicente del Caguán, Laureles, Resguardo Indígena Altamira, 917 m., 2°27'50.14"N; 74°55'02.06"W. Paratype female. Caquetá. Belén de Los Andaquíes, Resguardo Indígena La Esperanza, 844 m., 1°36'19.18"N: 75°56'12.46"W.

##### Type material.

Holotype male. 27.IV.2017. Led light trap. J. Panche. MUSENUV, slide code No. 28777. Paratype female, 1.III.2017. Led light trap. J. Panche. MUSENUV, slide code 28778).

##### Etymology.

The specific epithet refers to the Colombian department of Caquetá, where the types were collected.

##### Diagnosis.

Belonging in species group B, in the classification of García Aldrete et al. (2013). It is close to *E. daguaensis* González, García Aldrete & Carrejo and to *E. gaitanae* sp. n., described below; differing from them by having the lateral processes of the central sclerite of the hypandrium with abundant distal teeth and not bent distally inwards. Median projections separated from the basal part as in *E. daguaensis* (Fig. 4). Forewing with a pigmented marginal band, from cell R_3_ to wing base. Female IX sternum trapeziform, slightly convex anteriorly (Fig. 11).

##### Description.

**Figures 1–6.**
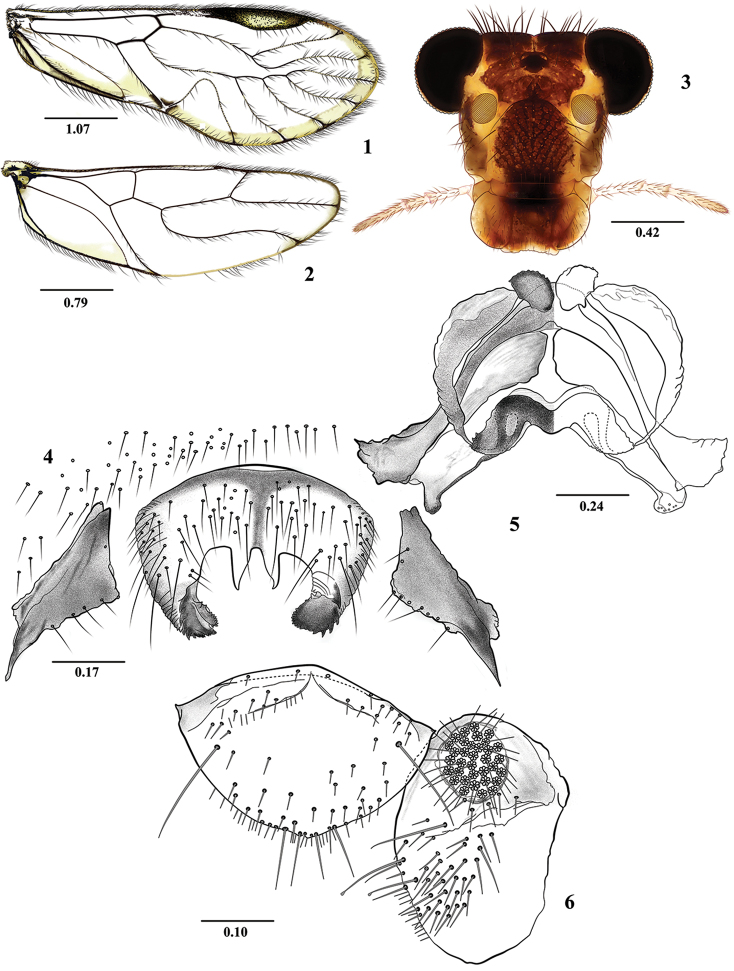
*Euplocania caquetaensis* sp. n. Male. **1** Forewing **2** Hindwing **3** Front view of head **4** Hypandrium **5** Phallosome **6** Epiproct and right paraproct. Scales in millimeters.

***Male.* Color** (in 80% ethanol). Head pattern (Fig. 3). Compound eyes black, ocelli hyaline, with ochre centripetal crescents. Frons, postclypeus, anteclypeus, and labrum dark brown centrally, with sides pale brown. Genae brown. Antennae creamy. Mx 1-3 creamy; Mx4 creamy, brown distally. Tergal lobes of meso- and metathorax dark brown. Thoracic pleura creamy, with small brown ochre spots. Coxae of all legs brown; femora of all legs pale brown; trochanters, tibiae and tarsi of all legs brown. Forewings almost hyaline, with a pale brown-yellowish band along margin, from R_2+3_ to near the wing base; veins brown, with a dark brown spot at wing margin. Pterostigma peripherally dark brown, pale brown in the middle (Fig. 1). Hindwings hyaline, veins brown, with a dark brown spot at end of R_4+5_ and M_1_ (Fig. 2). Abdomen creamy, with brown ochre spots. Central sclerite of hypandrium pale brown, basal and distal part of lateral processes dark brown. Epiproct and paraprocts creamy.

**Morphology.** As in diagnosis, plus the following: Head (Fig. 3): H/MxW: 1.36; compound eyes large, H/d: 2.54; IO/MxW: 0.69. Outer cusp of lacinial tip broad, with seven denticles. Mx4/Mx2: 1.21. Forewings (Fig. 1): L/W: 2.73. Pterostigma: lp/wp: 5.76, areola postica tall, with rounded apex: al/ah: 1.52. Hindwings (Fig. 2): l/w: 3.07. Central sclerite of hypandrium rounded anteriorly, triconcave posteriorly, side sclerites broadly triangular (Fig. 4). Phallosome (Fig. 5) anteriorly U shaped, with broad distally side struts; external parameres membranous, distally rounded, bearing pores; two pairs of endophallic sclerites, and one transverse mesal sclerite as illustrated. Paraprocts (Fig. 6) almost elliptic, with a dense setal field; sensory fields with 33 trichobothria on basal rosettes. Epiproct (Fig. 6) broad, convex anteriorly, with rounded apex and four apical macrosetae; setal field broad, with abundant small setae and two macrosetae, one on each side, as illustrated.

**Measurements.** FW: 5400, HW: 3650, f1: 1440, f2: 1520, f3: 1390, Mx4: 350, IO: 570, D: 500, d: 390, IO/d: 1.46, PO: 0.78.

***Female.* Color**. As in the male. Subgenital plate hyaline in the middle, with sides dark brown, as illustrated (Fig. 10).

**Figures 7–12.**
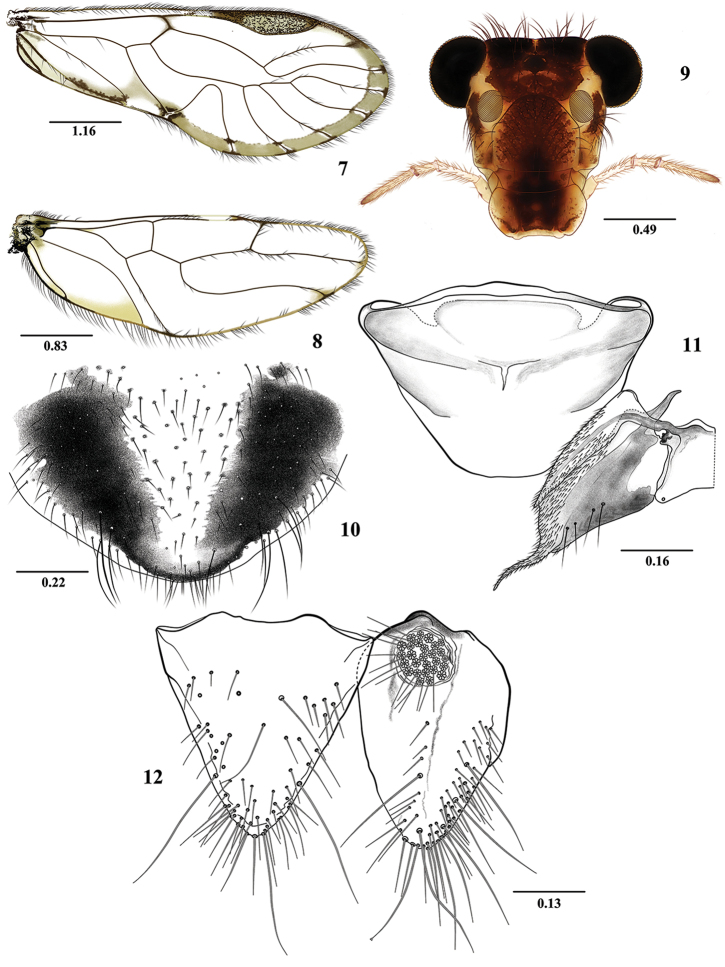
*Euplocania caquetaensis* sp. n. Female. **7** Forewing **8** Hindwing **9** Front view of head **10** Subgenital plate **11** Ninth sternum and left gonapophyses (ventral view) **12** Epiproct and right paraproct. Scales in millimeters.

**Morphology**. As in diagnosis, plus the following: Head (Fig. 9): H/MxW: 1.42; H/d: 3.30; IO/MxW: 0.70. Outer cusp of lacinial tip broad, with eight denticles. Mx4/Mx2: 1.25. Wings (Figs 7 and 8) as in the male, L/W: 2.65. Pterostigma: lp/wp: 5.33, areola postica: al/ah: 1.36. Hindwings (Fig. 8): l/w: 2.88. Subgenital plate (Fig. 10) broad, posteriorly rounded, setose. Gonapophyses (Fig. 11): v1 elongate, broad and pilose, acuminate; v2+3 pilose, with a row of 4 setae on v2; distal process sinuous, acuminate, with microsetae on surface. Paraprocts (Fig. 12) triangular, distal setal field with abundant setae as illustrated, sensory field with 23 trichobothria on basal rosettes. Epiproct (Fig. 12) triangular, mesal field with three macrosetae and abundant setae distally as illustrated.

**Measurements.** FW: 6125, HW: 4075, F: 1525, T: 2725, t1: 1100, t2: 105, t3: 175, ctt1: 36, f1: 1310, f2: 1440, f3: 1290, Mx4: 405, IO: 655, D: 500, d: 400, IO/d: 1.31, PO: 0.8.

#### Euplocania gaitanae sp. n.

Taxon classificationAnimaliaPsocodeaPtiloneuridae

http://zoobank.org/C434D2D3-9F04-408D-92DA-FEB3DE6E8C8E

Figs 13–18 , 19–24

##### Type locality.

COLOMBIA. Caquetá. Belén de Los Andaquíes, Resguardo Indígena La Esperanza, 844 m., 1°36'19.18"N; 75°56'12.46"W.

##### Type material.

Holotype male. 1.III.2017. Led light trap. J. Panche. MUSENUV slide code No. 28779. Paratypes: 2 males, 1 female. MUSENUV slide code 28780, same data as the holotype.

##### Etymology.

This species is dedicated to the female cacique Gaitana (Guaitipán), indigenous heroine of the XVI century, who led a ferocious resistance against the Spanish invaders in the mountains of the Huila-Caquetá Departments, in the Colombian Andes.

##### Diagnosis.

**Figures 13–18.**
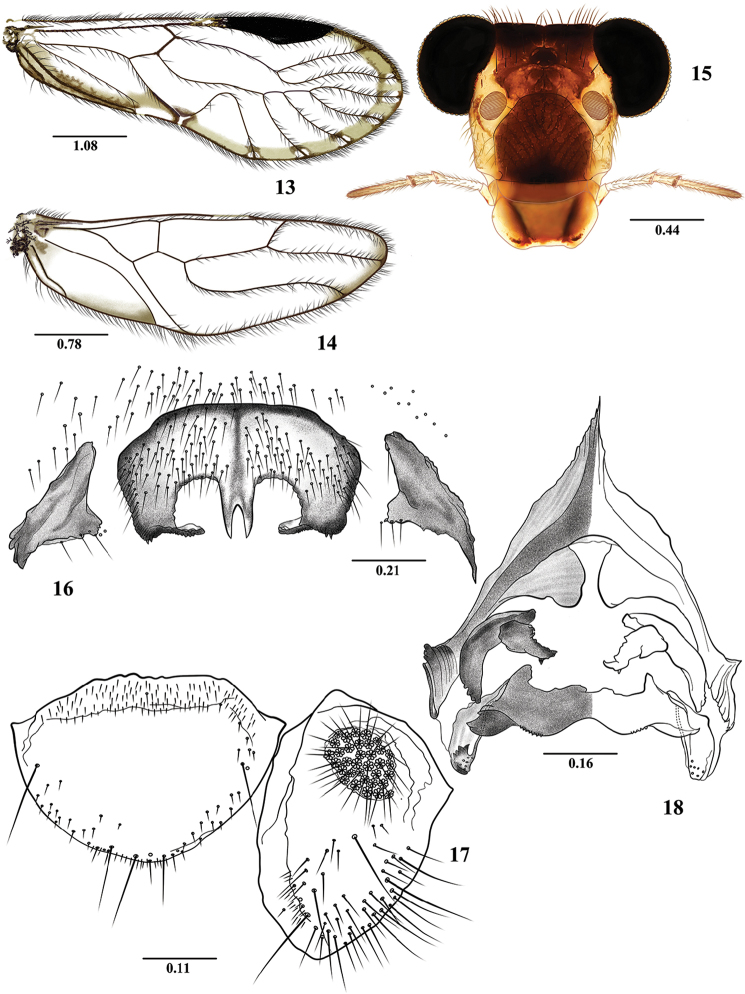
*Euplocania gaitanae* sp. n. Male. **13** Forewing **14** Hindwing **15** Front view of head **16** Hypandrium **17** Epiproct and right paraproct **18** Phallosome. Scales in millimeters.

**Figures 19–24.**
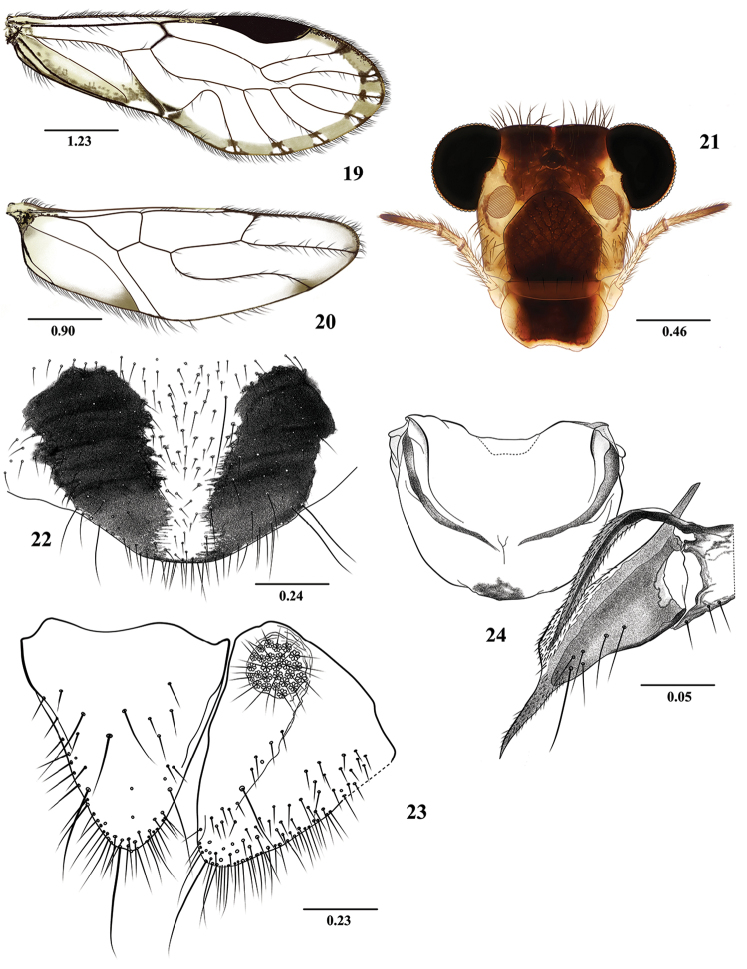
*Euplocania gaitanae* sp. n. Female. **19** Forewing **20** Hindwing 21. Front view of head **22** Subgenital plate **23** Epiproct and right paraproct **24** Ninth sternum and left gonapophyses (ventral view). Scales in millimeters.

Belonging in species group B, in the classification of García Aldrete et al. (2013). It is similar to *E. daguaensis* González, García Aldrete & Carrejo and to *E. caquetaensis* sp. n. It differs from them by the shape of the median and lateral processes of the central sclerite of the hypandrium and in details of phallosomes and forewings (see identification key above). Central sclerite of hypandrium wide, posteriorly with two short acuminate projections in the middle, and two lateral processes, bearing two short apical teeth, without teeth on the inner border, bent inwards (Fig. 16). Phallosome V shaped, with a large transverse mesal endophallic sclerite (Fig. 18). Forewings with a pigmented marginal band, from R_2+3_ to wing base (Figs 13 and 19). Female IX sternum semioval, anteriorly concave medially, sides with narrow pigmented area bent towards the mesal line (Fig. 24).

##### Description.

***Male.* Color.** Head dark brown frontally, pale brown laterally (Fig. 15). Compound eyes black, ocelli hyaline, with ochre centripetal crescents. Vertex, clypeus and labrum dark brown. Genae pale brown, with small ochre band near the antennal fossae. Antennae pale brown to brown, with apex cream. Maxillary palps brown, Mx4 distally dark brown. Tergal lobes of meso- and metathorax dark brown. Thoracic pleura creamy, with ochre and white spots. Legs brown, coxae dark brown. Forewings hyaline, with a pale brown marginal band, from R_2+3_ to wing base; veins brown, with a dark brown spot at wing margin. Pterostigma dark brown (Fig. 13). Hindwings hyaline, veins brown, with a pale brown spot at wing margin (Fig. 14). Abdomen creamy, with ochre subcuticular bands. Central sclerite of hypandrium pale brown, with sides dark brown. Epiproct and paraprocts creamy.

**Morphology.** As in diagnosis, plus the following: Head (Fig. 15): H/MxW: 1.48; compound eyes large, H/d: 2.77; IO/MxW: 0.64. Outer cusp of lacinial tip broad, with nine denticles. Mx4/Mx2: 1.11. Forewings (Fig. 13): L/W: 2.77. Pterostigma: lp/wp: 5.13, areola postica tall, with rounded apex: al/ah: 1.63. Hindwings (Fig. 14): l/w: 3.05. Central sclerite of hypandrium rounded anteriorly, triconcave posteriorly, side sclerites triangular (Fig. 16). Phallosome (Fig. 18) anteriorly Y shaped, with broad side struts; external parameres membranous, distally rounded, bearing pores; two pairs of endophallic sclerites, and one posterior, transverse mesal sclerite. Paraprocts (Fig. 17) almost elliptic, with a dense setal field distally; sensory fields with 29 trichobothria on basal rosettes. Epiproct (Fig. 17) broad, semioval, rounded posteriorly, setal field on sides and anteriorly; one large setae on each side and three macrosetae posteriorly.

**Measurements.** FW: 5750, HW: 3962, F: 1475, T: 2600, t1: 1057, t2: 100, t3: 150, ctt1: 36, f1: 1250, f2: 1390, f3: 1310, Mx4: 365, IO: 560, D: 600, d: 470, IO/d: 1.19, PO: 0.78.

***Female.* Color**. Essentially as in the male. Subgenital plate hyaline in the middle, with sides dark brown, as illustrated (Fig. 22).

**Morphology**. As in diagnosis, plus the following: Head (Fig. 21): H/MxW: 1.41; H/d: 3.09; IO/MxW: 0.68. Outer cusp of lacinial tip broad, with eight denticles. Mx4/Mx2: 1.25. Wings (Figs 19 and 20) as in the male, L/W: 2.76. Pterostigma: lp/wp: 4.71, areola postica: al/ah: 1.82. Hindwings (Fig. 20): l/w: 3.06. Subgenital plate (Fig. 22) broad, posteriorly rounded, setose. Gonapophyses (Fig. 24): v1 elongate, slender, setose, acuminate; v2+3 with a row of 6 setae on v2; distal process sinuous, acuminate, with microsetae on surface. Paraprocts (Fig. 23) triangular, with distal setal field as illustrated, sensory field with 27 trichobothria on basal rosettes. Epiproct (Fig. 23) triangular, apically rounded, setae as illustrated.

**Measurements.** FW: 6200, HW: 4250, F: 1550, T: 2700, t1: 1050, t2: 100, t3: 152.5, ctt1: 28, f1: 1320, f2: 1470, f3: 1360, Mx4: 390, IO: 640, D: 536, d: 430, IO/d: 1.19, PO: 0.80.

#### Euplocania laelsa sp. n.

Taxon classificationAnimaliaPsocodeaPtiloneuridae

http://zoobank.org/1BF3832A-065D-4D72-8DFF-47B5AEE67301

Figs 25–30 , 31–36

##### Type locality.

COLOMBIA. Valle del Cauca. Dagua, La Elsa, Finca La Elsa, 942 m., 03°34'18.9"N; 76°45'46"W. Paratypes: 2 females, 2 males. Same data as the holotype.

##### Type material.

Holotype male. 7.IV.2017. Shannon light trap. J. S. Ramírez and R. González. MUSENUV, slide code No. 28781. Paratypes: 1 female, MUSENUV slide code 28782, 2 males, 1 female, same locality, 21.IV.2017. Shannon light trap. A. F. Vinasco and R. González. MUSENUV.

##### Etymology.

The specific name, a noun in apposition, refers to the type locality, Finca La Elsa, where the types were found.

##### Diagnosis.

**Figures 25–30.**
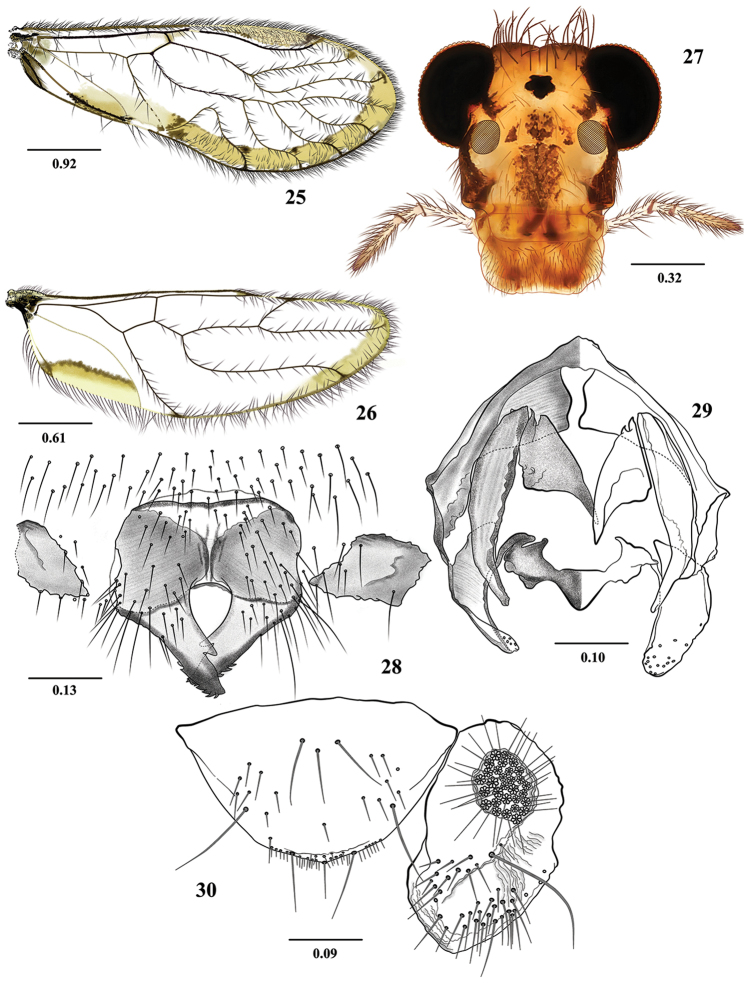
*Euplocania laelsa* sp. n. Male. **25** Forewing **26** Hindwing **27** Front view of head **28** Hypandrium **29** Phallosome **30** Epiproct and right paraproct. Scales in millimeters.

Belonging to the new species group *Laelsa*. Forewings with a broad, pigmented marginal band from R_4+5_ to Cu2-1A. Pterostigma elongate, not angulated towards Rs (Fig. 25). Hypandrium of three sclerites, central one large, almost rectangular, with two stout lateral posterior processes, distally crossed, each bearing a mesal tooth on inner border, and a row of teeth distally along the outer border (Fig. 28). The sclerite above is reminiscent of the central sclerite of the hypandrium of *E. reyesi* García Aldrete, González & Carrejo (Group *Zelayensis*), but in the latter the posterior processes are smooth, the forewings are hyaline, and the phallosome is distinct, lacking a transverse mesal sclerite (Fig. 29).

##### Description.

***Male.* Color** (in 80% ethanol). Body pale brown, with creamy areas and brown ochre spots, as indicated below. Head frontally creamy, with ochre areas as illustrated (Fig. 27), genae ochre. Compound eyes black, ocelli hyaline, with ochre centripetal crescents. Antennae pale brown, flagellomeres 1-4 cream, flagellomeres 5-11 pale brown proximally and cream distally. Maxillary palps brown, Mx4 with distal third dark brown. Tergal lobes of meso- and metathorax dark brown. Thorax: mesepimeron dark brown, pro- and metapleura cream, with ochre spots. Legs: fore- and middle brown; hind coxae, trochanter and femur cream, hind tibia and tarsi pale brown. Forewings with pigmented marginal band, from R_2+3_ to near the wing base, veins brown, with a dark brown spot at wing margin. Pterostigma peripherally pale, brown-yellowish in the middle (Fig. 25). Hindwings hyaline, veins brown, with a pigmented marginal band on apex and near the wing base (Fig. 26). Abdomen cream, with broad subcuticular ochre spots. Clunium and hypandrium pale brown, lateral processes of the central sclerite of hypandrium dark brown apically. Epiproct and paraprocts cream, with ochre spots; phallosome pale brown.

**Morphology.** As in diagnosis, plus the following: Head (Fig. 27): Vertex with abundant setae. H/MxW: 1.56; compound eyes large, H/d: 3.12; IO/MxW: 0.57. Outer cusp of lacinial tip broad, with six denticles. Mx4/Mx2: 1.17. Forewings (Fig. 25): L/W: 2.67. Pterostigma: lp/wp: 6.09, areola postica tall, with rounded apex: al/ah: 2.05. Hindwings (Fig. 26): l/w: 2.98. Hypandrium of three sclerites (Fig. 28). Phallosome (Fig. 29) anteriorly U shaped, with distally broad side struts; external parameres membranous, distally rounded, bearing pores; two pairs of endophallic sclerites, and one transverse mesal sclerite as illustrated; mesal sclerite with posterior central projection triangular. Paraprocts (Fig. 30) almost elliptic, with a dense setal field; sensory fields with 26 trichobothria on basal rosettes. Epiproct (Fig. 30) broad, semi-oval, with rounded apex and three apical macrosetae, mesal field with abundant small setae, two macrosetae, one on each side and central field with three macrosetae as illustrated.

**Figures 31–36.**
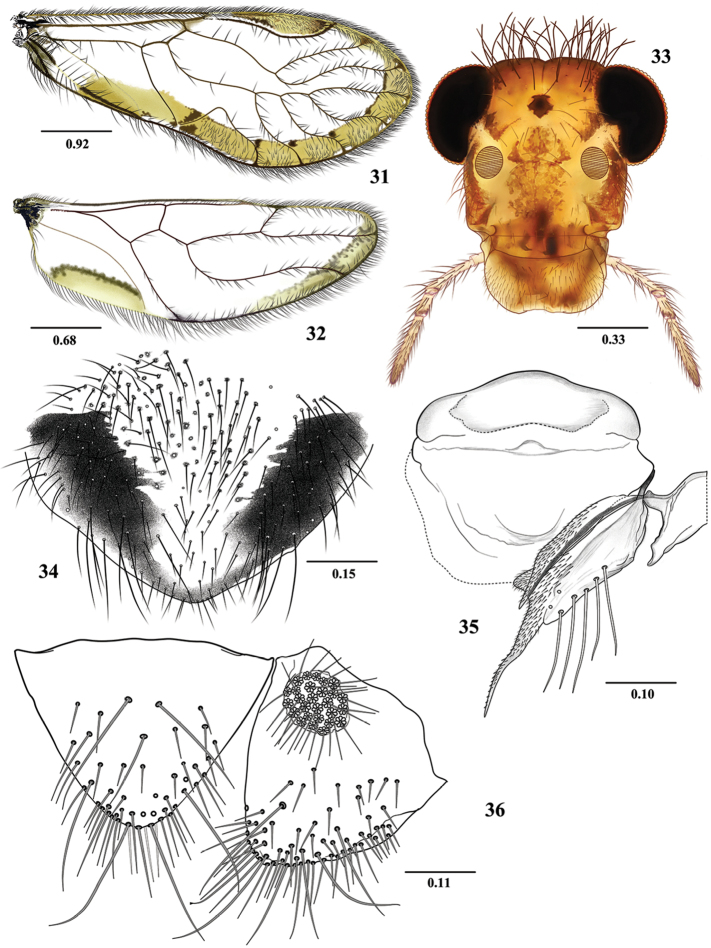
*Euplocania laelsa* sp. n. Female. **31** Forewing **32** Hindwing **33** Front view of head **34** Subgenital plate **35** Ninth sternum and left gonapophyses (ventral view) **36** Epiproct and right paraproct. Scales in millimeters.

**Measurements.** FW: 4800, HW: 3125, F: 1150, T: 2025, t1: 825, t2: 80, t3: 157, ctt1: 23, f1: 880, f2: 750, f3: 690, f4: 620, f5: 440, f6: 420, f7: 360, f8: 310, f9: 280, f10: 230, f11: 260, Mx4: 280, IO: 386, D: 470, d: 340, IO/d: 1.14. PO: 0.72.

***Female.* Color**. As in the male. Subgenital plate hyaline in the middle, with sides pale brown, as illustrated (Fig. 34).

**Morphology**. As in diagnosis, plus the following: Head (Fig. 33): vertex with abundant large setae. H/MxW: 1.52; H/d: 3.25; IO/MxW: 0.61. Outer cusp of lacinial tip broad, with six denticles. Mx4/Mx2: 1.33. Wings (Figs 31 and 32) as in the male, L/W: 2.56. Pterostigma: lp/wp: 5.08, areola postica: al/ah: 2.09. Hindwings (Fig. 32): l/w: 2.98. Subgenital plate (Fig. 34) broad, posteriorly rounded, setose. Gonapophyses (Fig. 35): v1 elongate, pilose, acuminate; v2+3, pilose, with a row of five macrosetae on v2; distal process sinuous, acuminate, with microsetae on surface. Paraprocts (Fig. 36) broadly triangular, distal setal field with abundant setae as illustrated, sensory fields with 26 trichobothria on basal rosettes. Epiproct (Fig. 36) triangular, mesal field with three macrosetae, distal field with abundant setae as illustrated.

**Measurements.** FW: 5250, HW: 3500, F: 1250, T: 2125, t1: 837, t2: 92, t3: 137, ctt1: 27, f1: 930, f2: 870, f3: 800, f4: 690, Mx4: 350, IO: 470, D: 480, d: 360, IO/d: 0.98, PO: 0.75.

#### Euplocania nasa sp. n.

Taxon classificationAnimaliaPsocodeaPtiloneuridae

http://zoobank.org/A97EB2A8-BB13-4CAD-A3C1-DB154E15D8CD

Figs 37–42 Male

##### Type locality.

COLOMBIA. Huila. Acevedo, National Natural Park Los Guácharos, 1882 m., 1°36'45.9"N; 76°06'15.4"W.

##### Type material.

Holotype male. 31.VII.2016. On tree trunk. N. Carrejo, R. González & J. Mendivil. MUSENUV, slide code No. 28783.

##### Etymology.

This species is dedicated to the Nasa tribe, that inhabits a wide region of mountains in the departments of Huila and Caquetá. The name is a noun in apposition.

##### Diagnosis.

**Figures 37–42.**
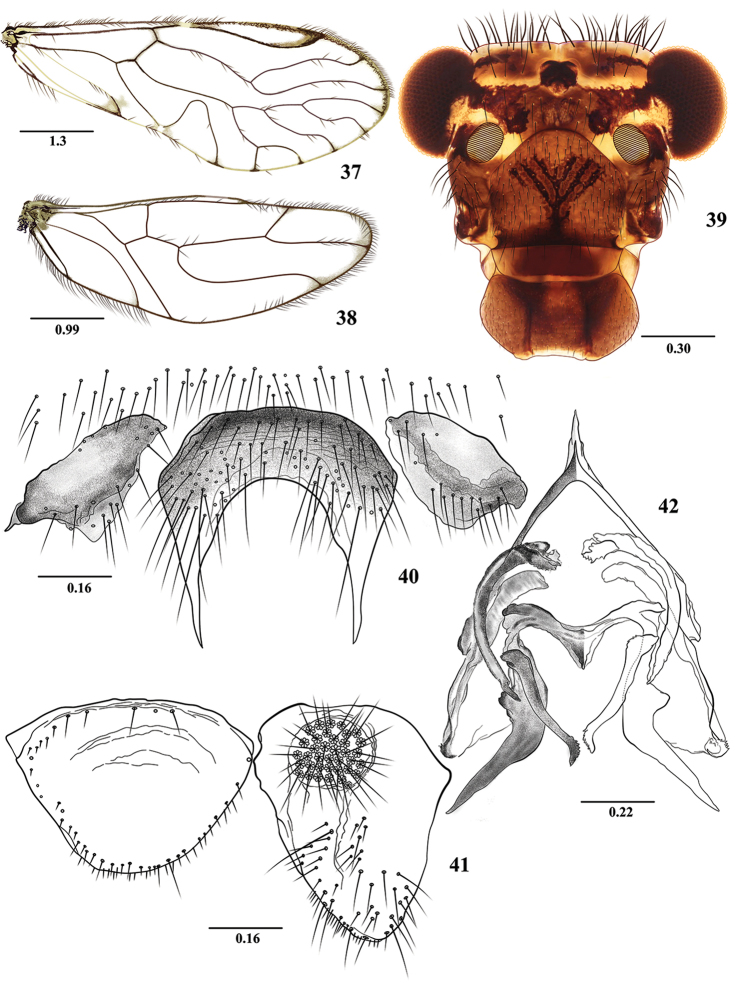
*Euplocania nasa* sp. n. Male. **37** Forewing **38** Hindwing **39** Front view of head **40** Hypandrium **41** Epiproct and right paraproct **42** Phallosome. Scales in millimeters.

Belonging to the new species group *Guentherbuchi*. Forewings hyaline. Pterostigma elongate, not angulated towards Rs (Fig. 37). Hypandrium of three sclerites, central one rounded anteriorly, with two lateral, slender, elongate, acuminate posterior processes (Fig. 40). Related to *E. guentherbuchi* González, García Aldrete & Carrejo, differing from it by having the posterior processes of the central sclerite of the hypandrium stouter and much broader proximally. The phallosomes in both species are built on the same plan, but differ in details of the endophallic sclerites (compare Fig. 42 in this paper with fig. 50 in González-Obando et al. 2015).

##### Description.

**Color** (in 80% ethanol). Body brown. Head dark brown with small areas cream (Fig. 39). Compound eyes black, ocelli hyaline, with ochre centripetal crescents. Antennae brown, flagellomeres pale brown, with apices cream. Maxillary palps pale brown, Mx4 with distal third dark brown. Tergal lobes of meso- and metathorax brown. Thoracic mesopleura brown, mesepisternum more pigmented. Legs: fore- and hind- coxae creamy, with small proximal and distal brown spots; mid coxae brown, trochanters and femora creamy, tibia, tarsi and apex of femora brown. Wings hyaline, veins brown, vein ends more pigmented as illustrated (Figs 37 and 38). Abdomen creamy, with subcuticular, transverse ochre bands. Clunium, hypandrium, epiproct and paraprocts pale brown, phallosome brown, with endophallic sclerites more pigmented.

**Morphology.** As in diagnosis, plus the following: Head (Fig. 39): H/MxW: 1.40, H/d: 3.86, compound eyes large: IO/MxW: 0.78. Vertex slightly concave in the middle. Outer cusp of lacinial tip broad, with seven denticles. Mx4/Mx2: 1.23. Forewings (Fig. 37) with M four-branched; M4 distally forked, L/W: 2.56, pterostigma elongate: lp/wp: 6.0; areola postica tall, slightly slanted posteriorly, apex rounded, al/ah: 1.30. Hindwing (Fig. 38): l/w: 2.74. Hypandrium of three sclerites, the central one abundantly setose, convex anteriorly, with a deep concavity posteriorly; side sclerites elongate, broadly triangular (Fig. 40). Phallosome anteriorly Y-shaped (Fig. 42), external parameres distally rounded, bearing pores; anterior endophallic sclerites curved, distally acuminate, antero-central sclerites small, denticulate. Mesal endophallic sclerites transverse, with meso-posterior projection triangular, postero-mesal sclerite with a rounded protuberance basally, each arm dilated basally, bent outwards, distally acuminate; posterior pair elongate, directed inwards, distally bent outwards, denticulate. Paraprocts (Fig. 41) robust, elongate, setose as illustrated, sensory fields with 32 trichobothria on basal rosettes. Epiproct wide, semioval, rounded posteriorly, straight anteriorly, setae as illustrated (Fig. 41).

**Measurements.** FW: 6900, HW: 4625, F: 1725, T: 3050, t1: 1275, t2: 100, t3: 167, ctt1: 35, f1: 1450, f2: 1570, f3: 1390, Mx4: 395, IO: 690, D: 460, d: 324, IO/d: 2.13, PO: 0.70.

#### Euplocania yalcona sp. n.

Taxon classificationAnimaliaPsocodeaPtiloneuridae

http://zoobank.org/5B714FD9-44D7-43FE-8374-D699447F4664

Figs 43–48 Male

##### Type locality.

COLOMBIA. Huila. Palestina, El Encanto Nature Reserve, 1462 m., 1°43'10.3"N; 76°07'1.7"W.

##### Type material.

Holotype male. 29.VII.2016. On rock surfaces. J. Mendivil & R. González. MUSENUV slide code No. 28784.

##### Etymology.

The specific epithet (feminine form of the adjective *yalconus, -a, -um*) refers to the Yalcon indigenous people, who inhabited the Upper Magdalena Valley, in the Department of Huila, Colombia.

##### Diagnosis.

**Figures 43–48.**
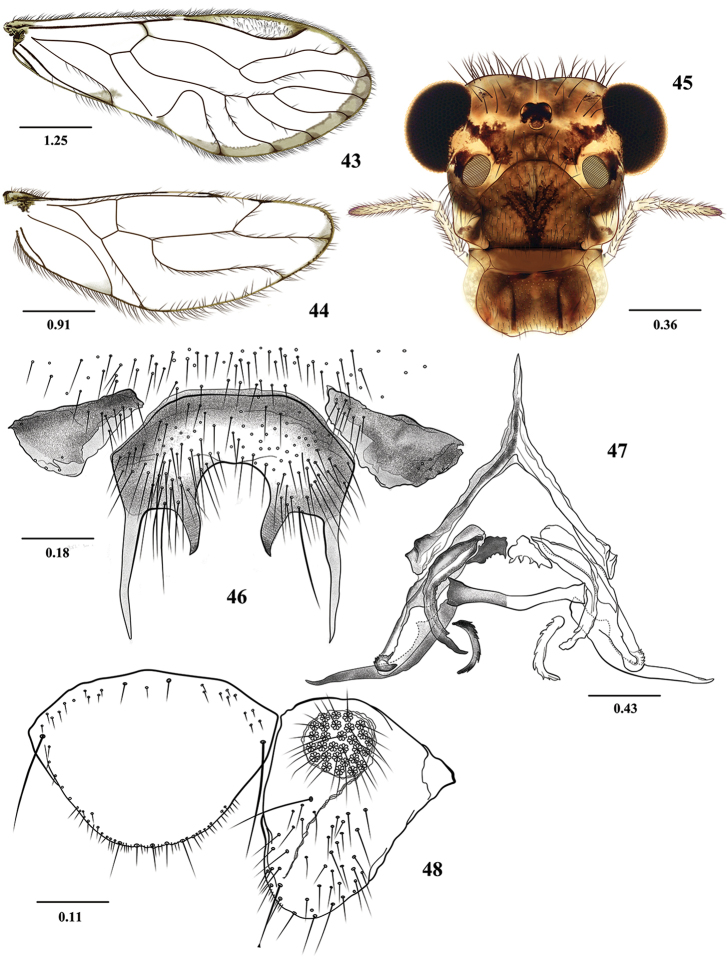
*Euplocania yalcona* sp. n. Male. **43** Forewing **44** Hindwing **45** Front view of head **46** Hypandrium **47** Phallosome **48** Epiproct and right paraproct. Scales in millimeters.

Belonging to the new species group *Yalcona*. Forewings with a slender, pigmented marginal band, from R_4+5_ to areola postica (Fig. 43). Pterostigma elongate, not angulated towards Rs. Hypandrium of three sclerites, central one anteriorly straight, with two lateral, long, slender acuminate posterior processes, and two median, shorter, acuminate posterior processes (Fig. 46). Phallosome built on the same plan as in species group *Guentherbuchi*, but differing in details of the endophallic sclerites.

##### Description.

***Male.* Color** (in 80% ethanol). Body pale brown, with creamy areas, as indicated below. Head creamy, with dark brown areas as illustrated (Fig. 45). Compound eyes black, ocelli hyaline, with ochre centripetal crescents. Antennae pale brown, flagellomeres with apices cream. Maxillary palps pale brown, Mx4 dark brown apically. Tergal lobes of meso- and metathorax dark brown. Thoracic mesopleura brown, more pigmented than pro- and metapleura. Legs: fore- and hind- coxae creamy, with small proximal and distal brown spots; mid coxae brown, trochanters and femora pale brown, tibia and tarsi brown. Wings almost hyaline, forewings as diagnosed above (Figs 43 and 44). Abdomen creamy, with subcuticular transverse ochre bands. Clunium and hypandrium brown. Epiproct and paraprocts pale brown, phallosome pale brown, with endophallic sclerites more pigmented.

**Morphology.** As in diagnosis, plus the following: Head (Fig. 45): H/MxW: 1.44, H/d: 3.70, compound eyes large: IO/MxW: 0.73. Vertex slightly concave in the middle. Outer cusp of lacinial tip broad, with five denticles. Mx4/Mx2: 1.09. Forewings (Fig. 43) with M four-branched; M4 simple, paratypes often with M of 5 branches, L/W: 2.58, pterostigma elongate: lp/wp: 5.63; areola postica tall, slightly slanted posteriorly, apex rounded, al/ah: 1.66. Hindwings (Fig. 44): l/w: 2.70. Hypandrium (Fig. 46). Phallosome anteriorly Y-shaped (Fig. 47), external parameres membranous, distally rounded, bearing pores; anterior endophallic sclerites curved, distally acuminate, central sclerite short, denticulate. Mesal endophallic sclerites transverse, postero-mesal sclerite curved outwards, distally acuminate, each arm with a rounded protuberance proximally; posterior pair slender, denticulated, and bent outwards. Paraprocts (Fig. 48) robust, elongate, setose as illustrated, sensory fields with 32 trichobothria on basal rosettes. Epiproct wide, semioval, posteriorly rounded, convex anteriorly, with macrosetae on each basal angle, posterior border with setae as illustrated (Fig. 48).

**Measurements.** FW: 6325, HW: 4125, F: 1600, T: 2675, t1: 1162, t2: 105, t3: 175, ctt1: 34, f1: 1320, f2: 1450, f3: 1240, f4: 1130, f5: 690, f6: 630, f7: 490, f8: 390, Mx4: 350, IO: 620, D: 450, d: 330, IO/d: 1.88, PO: 0.73.

#### New Colombian records

***Euplocania badonneli* New & Thornton, 1988** 1 male. Amazonas, Leticia, San Martín de Amacayacú. Agua Blanca trail, 3°42'19.0"S; 70°20'26.1"W, 70 m. 12-13.VIII.2015. R. González, N. Carrejo, N. Calderón, O. Saenz. Led light trap on forest canopy. 1 male. Caquetá, Jericó-Consayá, La Raya trail, 0°33'21.18"N; 75°05'15.57"W, 201 m. 29.IX.2016, J. Panche. Led light trap. 1 male. Caquetá, San Vicente del Caguán, Laureles, Resguardo Indigena Altamira, 2°27'50.14"N; 74°55'02.06"W, 917 m. 26.IV.2017, J. Panche. Led light trap. 1 male. Putumayo, Puerto Asis, Las Delicias, 0°22'09.50"N; 76°31'01.98"W, 264 m. 5.III.2014. J. Panche, led light trap.

## Discussion

The species of *Euplocania* here dealt with, have increased the spectrum of morphological variability within the genus, making necessary the creation of three additional species groups, modifying the initial scheme presented by García Aldrete et al. (2013), diagnosed as follows:

**Group *Guentherbuchi*.** Forewings hyaline. Pterostigma elongate, not angulated towards Rs (Fig. 37). Hypandrium of three sclerites, central one rounded anteriorly, with two lateral, slender, elongate, acuminate posterior processes (Fig. 40). Mesal endophallic sclerites transverse, with meso-posterior projection, postero-mesal sclerite with a protuberance basally, each arm dilated proximally, bent outwards, distally acuminate (Fig. 42). *E. guentherbuchi* had been assigned in species group *Zelayensis*, but it does not belong there and constitutes a different species group. Species included: *E. guentherbuchi* González et al. (2015), *E. nasa* sp. n.

**Group *Laelsa*.** Forewings with a broad, pigmented marginal band from R_4+5_ to Cu2-1A. Pterostigma elongate, not angulated towards Rs (Fig. 25). Hypandrium of three sclerites, central one large, almost rectangular, with two stout lateral posterior processes, distally crossed, each bearing a mesal tooth on inner border, and a row of teeth distally along the outer border (Fig. 28). Phallosome with two pairs of endophallic sclerites one transverse mesal with posterior central projection (Fig. 29). Species included: *E. laelsa* sp. n.

**Group *Yalcona*.** Forewings with a slender, pigmented marginal band, from R_4+5_ to areola postica (Fig. 43). Pterostigma elongate, not angulated towards Rs. Hypandrium of three sclerites, central one anteriorly straight, with two lateral, long, slender acuminate posterior processes, and two median, shorter, acuminate posterior processes (Fig. 46). Phallosome with mesal endophallic sclerites transverse, postero-mesal sclerite distally acuminate, each arm with a rounded protuberance proximally (Fig. 47). Species included: *E. yalcona* sp. n.

The species here treated raise to 22 the species known in the genus, and raise to 14 the species of the genus known in Colombia (Table 1), one of them shared with Brazil and Peru; the rest are possibly endemic to this country. Many undescribed species of *Euplocania* from South America are known, already available for study in our collections; they will be dealt with in the near future, they increase the number of Colombian species to 30 (16 undescribed); Brazil has 13 species but to date only four species have been described, Ecuador has 14 species, but none has been described. Their study will bring additional modifications to the classification of the genus proposed by García Aldrete et al. (2013) and it will be necessary to conduct a phylogenetic analysis to verify its monophyly, as well as its relationships with other genera of the family Ptiloneuridae.

## Supplementary Material

XML Treatment for Euplocania caquetaensis

XML Treatment for Euplocania gaitanae

XML Treatment for Euplocania laelsa

XML Treatment for Euplocania nasa

XML Treatment for Euplocania yalcona
